# Imaging characteristics of gastrointestinal foreign bodies in children and analysis of risk factors associated with the severity of complications

**DOI:** 10.3389/fped.2026.1857502

**Published:** 2026-06-15

**Authors:** Hongzhi Du, Liling Shi

**Affiliations:** Department of Ultrasound, Shanxi Children's Hospital & Shanxi Women and Children Health Hospital, Taiyuan, China

**Keywords:** children, complications, gastrointestinal foreign body, imaging, magnetic beads, risk factors

## Abstract

**Objective:**

To analyze the clinical characteristics, imaging findings, and risk factors for complications of gastrointestinal foreign bodies in children, and to provide evidence for clinical diagnosis and treatment.

**Methods:**

A retrospective analysis was conducted on 78 foreign body retention sites in 71 pediatric patients diagnosed with gastrointestinal foreign bodies in a hospital from August 2020 to December 2025. Data including demographic characteristics, types of foreign bodies, imaging findings, complications, and treatment methods were collected. Foreign bodies were classified into six categories. Retention time was defined as the interval from the confirmed or most suspected time of ingestion to the time of imaging examination. Interobserver agreement for ultrasound interpretation was assessed using Cohen's *κ* between two senior radiologists. Univariate and multivariate logistic regression analyses were performed to identify independent risk factors for complications.

**Results:**

Among 71 children, 57.7% were male; median age 3 years. Magnetic beads were most common (54.9%), followed by coins (12.7%) and water-absorbing beads (8.5%). The small intestine was the main impaction site (41.5%). Complications occurred in 50 patients (70.4%): perforation in 32 (45.1%) and obstruction in 26 (36.6%). Magnetic beads caused perforation in 71.8%; water beads caused obstruction in 100%. Ultrasound effectively detected radiolucent foreign bodies, whereas plain radiography did not. Independent risk factors for complications included sharp/corrosive foreign bodies (OR = 5.12), retention time >24 h (OR = 4.87), upper gastrointestinal location (OR = 3.21), and symptomatic presentation (OR = 3.94) (all *P* < 0.01). Magnetic beads independently predicted complications (OR = 6.43, *P* < 0.001). Surgery was required in 69.0%, endoscopy in 12.7%, and spontaneous passage in 18.3%. No deaths occurred.

**Conclusion:**

Magnetic beads are the most common and high-risk gastrointestinal foreign bodies in children. Ultrasound is valuable for detecting radiolucent objects. Retention time >24 h, upper gastrointestinal location, and symptoms warrant early intervention.

## 1 Introduction

Gastrointestinal foreign bodies refer to various indigestible objects that remain in the digestive tract without being expelled in time. Due to their strong curiosity, active exploratory behavior, and immature cognitive judgment, children often place non-food items into their mouths, making ingestion of foreign bodies a common occurrence in pediatric emergency settings. Epidemiological data worldwide indicate that children under 6 years old are the most affected group, with a peak incidence between 1 and 3 years of age (1). Although most gastrointestinal foreign bodies (such as coins and small toy parts) can pass through the gastrointestinal tract spontaneously without symptoms or intervention, certain types possess high pathogenic potential and may lead to serious complications within hours to days. These complications include mucosal erosion, ulceration, perforation, intestinal obstruction, and even life-threatening gastrointestinal hemorrhage or mediastinal/abdominal infections (2–4).

Historically, the majority of ingested foreign bodies in children have been reported to pass through the gastrointestinal tract without complications, with observational studies showing that over 80% of cases resolve spontaneously and only approximately 1% require surgical intervention (5). However, the epidemiological landscape of pediatric foreign body ingestion has changed substantially in recent years worldwide (6). In recent years, with the rapid evolution of consumer products, the variety of novel foreign bodies has increased significantly. Their physicochemical properties and biocompatibility vary greatly, and the associated clinical risks often exceed those of traditional inert objects. For instance, ingestion of multiple high-powered magnetic toys (such as magnetic beads) can result in mutual attraction across adjacent bowel loops, leading to pressure necrosis, ischemia, and perforation. Button batteries lodged in the esophagus can cause deep chemical burns reaching the muscular layer within as little as 2 hours due to electrochemical reactions. Meanwhile, water-absorbing polymers (such as “water beads”) can rapidly expand within the body and induce complete intestinal obstruction (7–9). Although the overall incidence of complications caused by these foreign bodies is relatively low, once they occur, disease progression can be rapid, with a markedly increased risk of disability or death.

At present, clinical management strategies largely rely on early imaging localization and individualized risk stratification. However, different types of foreign bodies exhibit markedly different imaging characteristics across commonly used modalities. Metallic objects (such as coins and magnets) are easily detectable on x-ray due to their radiopacity, whereas plastic, wood, rubber, and most biopolymeric materials (such as water beads) are radiolucent and difficult to identify on routine chest and abdominal radiographs, increasing the risk of missed diagnosis or misinterpretation (10). Although ultrasound demonstrates some sensitivity for detecting non-metallic foreign bodies, its accuracy is highly operator-dependent and may be affected by intestinal gas, and standardized diagnostic criteria are still lacking. Recent studies have reported that ultrasound can achieve a sensitivity of 90.65% and specificity of 100% for detecting gastrointestinal magnets in children, with satisfactory performance for localization in the stomach (96.30%) and small intestine (100.00%) (11). Furthermore, current clinical guidelines are largely based on expert consensus or small retrospective studies. There remains a lack of robust real-world, multivariate evidence to clarify which clinical or imaging factors—such as foreign body type, size, number, location, duration of retention, and patient age—independently predict the occurrence of complications.

Based on 71 representative cases treated in our hospital in recent years, this study integrates imaging characteristics with clinical outcomes to comprehensively analyze the independent risk factors for complications. The aim is to provide empirical evidence for establishing a more precise risk assessment model in clinical practice.

## 2 Materials and methods

### 2.1 Study population

This was a single-center retrospective cohort study. A total of 71 pediatric patients diagnosed with gastrointestinal foreign bodies at the pediatric emergency department or gastroenterology department of our hospital between August 2020 and December 2025 were included.

Inclusion criteria: (1) age <12 years; (2) a clear history of foreign body ingestion or highly suspected clinical history (e.g., sudden dysphagia, drooling, abdominal pain); (3) presence of a gastrointestinal foreign body confirmed by at least one imaging modality (x-ray, ultrasound, or CT) or endoscopy; (4) complete clinical data and follow-up records.

Exclusion criteria: (1) patients who left the hospital without completing evaluation and were lost to follow-up, making outcome assessment impossible; (2) foreign bodies that had been spontaneously expelled before presentation without any medical intervention or imaging confirmation. (3) patients with known gastrointestinal malformations that could independently predispose to complications.

This retrospective study was approved by the Institutional Review Board of Children's Hospital of Shanxi & Women Health Center of Shanxi. The requirement for informed consent was waived due to the retrospective nature of the study, the use of pre-existing anonymized data, and the absence of any interventions. The study was conducted in accordance with the Declaration of Helsinki and followed the Strengthening the Reporting of Observational Studies in Epidemiology (STROBE) reporting guideline.

### 2.2 Data collection

The following variables were extracted from the electronic medical record system:

Demographic data: sex and age (recorded in years and categorized into <3 years, 3–6 years, and >6 years for analysis);

Clinical characteristics: chief complaints (e.g., dysphagia, vomiting, abdominal pain, feeding refusal, asymptomatic, etc.);

Foreign body characteristics: type, number, maximum diameter (mm), and shape (regular/irregular);

Location of retention: classified based on imaging or endoscopic findings into stomach, duodenum, small intestine (jejunum/ileum), and colon;

Imaging modalities and findings: including the use and positive findings of x-ray, ultrasound, CT, and gastroscopy/enteroscopy;

Retention time: defined as the interval from the confirmed or most suspected time of ingestion to the time of imaging examination, dichotomized at the 24-hour threshold based on previous literature (3);

Complications: defined as pathological conditions requiring additional intervention or prolonged hospitalization, including but not limited to gastrointestinal perforation (confirmed by imaging or intraoperatively), intestinal obstruction (supported by clinical and imaging findings), corrosive injury (mucosal necrosis/ulceration under endoscopy), peritonitis (fever, peritoneal irritation signs with ascites or free air), fistula formation, and major bleeding (hemoglobin decrease >20 g/L or requiring transfusion);

Treatment methods: conservative observation (follow-up only), endoscopic removal, and surgical intervention. Foreign bodies were classified into six categories according to international consensus and previous literature (2, 3): (1) blunt harmless objects (e.g., coins, glass beads, plastic toys); (2) sharp objects (e.g., needles, blades, jujube pits, fish bones); (3) corrosive objects (mainly button batteries, occasionally other chemicals); (4) magnetic objects (including ≥1 high-strength magnet, such as magnetic beads or neodymium magnets); (5) expandable objects (water-absorbing polymers, such as “water beads”); (6) mass-forming objects (e.g., trichobezoars, aggregated drug packaging).

### 2.3 Imaging evaluation protocol

All children with suspected gastrointestinal foreign bodies routinely underwent both x-ray and ultrasound examinations, with the order adjusted according to the suspected type of foreign body. For metallic foreign bodies (e.g., magnetic beads, coins), anteroposterior and lateral chest–abdominal x-rays (kVp 70–90, mAs 2–5) were performed first for rapid localization. For non-metallic foreign bodies (e.g., water beads, plastic objects), or when the foreign body type was clearly reported by caregivers, abdominal ultrasound was performed first. Regardless of the initial modality, both examinations were typically combined for cross-verification.

x-ray images were independently reviewed by two senior radiologists, focusing on the location, number, morphology of the foreign body, and the presence of the “beads-on-a-string sign” (suggestive of multiple magnets in adjacent bowel loops). Ultrasound was performed using a high-frequency linear probe (7–12 MHz), with emphasis on identifying the “comet-tail artifact” (specific for magnetic beads) or well-defined anechoic cystic structures (suggestive of expandable foreign bodies). Interobserver agreement between the two radiologists for ultrasound interpretation was assessed using Cohen's *κ* coefficient. A *κ* value ≥0.75 was considered excellent agreement, 0.40–0.75 fair to good agreement, and <0.40 poor agreement.

CT scanning (128-slice spiral CT, slice thickness 1.5 mm) was reserved for the following conditions: (1) suspected perforation or intra-abdominal infection; (2) unclear localization on x-ray or ultrasound; (3) complex distribution of multiple foreign bodies. Endoscopy (primarily gastroscopy, with enteroscopy when necessary) was performed by experienced pediatric endoscopists and served as the diagnostic gold standard, with simultaneous foreign body removal when feasible.

The sensitivity and specificity of ultrasound versus plain radiography for detecting different types of foreign bodies were calculated using endoscopy or intraoperative findings as the reference standard. For magnetic foreign bodies, detection performance was further stratified by anatomical location (stomach, small intestine, colon).

### 2.4 Statistical analysis

Statistical analysis was performed using SPSS version 26.0.

Descriptive statistics: categorical variables were expressed as frequency (percentage); continuous variables were tested for normality using the Shapiro–Wilk test. Normally distributed data were expressed as mean ± standard deviation ($\bar{\times }$ ± s), while non-normally distributed data were expressed as median (P25–P75).

Univariate analysis: severe complications (defined as perforation, complete intestinal obstruction, peritonitis, deep corrosive ulceration, or death) were used as the dependent variable. Chi-square test, Fisher's exact test (when expected counts <5), or Mann–Whitney *U*-test were applied to evaluate demographic, clinical, and foreign body-related variables.

Multivariate analysis: variables with *P* < 0.1 in univariate analysis were included in a binary logistic regression model, and independent risk factors were identified using the forward likelihood ratio (Forward LR) method. Two separate multivariate models were constructed: Model 1 included the composite variable of “sharp or corrosive foreign bodies” (as in the original analysis), while Model 2 substituted this with “magnetic beads” as an independent categorical predictor to assess their specific risk. Model fit was assessed using the Hosmer–Lemeshow goodness-of-fit test, with *P* > 0.05 indicating acceptable fit. Results were expressed as odds ratios (ORs) with 95% confidence intervals (CIs).

A two-sided *P* value < 0.05 was considered statistically significant.

## 3 Results

### 3.1 General characteristics

A total of 71 children with gastrointestinal foreign bodies were included in this study, comprising 41 males (57.7%) and 30 females (42.3%). The age ranged from 11 months to 12 years, with a median age of 3 years (IQR: 1–5 years). According to age distribution, there were 2 cases (2.8%) aged <1 year, 36 cases (50.7%) aged 1–3 years, 19 cases (26.8%) aged 4–6 years, and 14 cases (19.7%) aged ≥7 years (Table 1).

**Table 1:** General demographic characteristics of patients (*n* = 71).

Variable	*n* (%) or median (IQR)
Sex	
Male	41 (57.7%)
Female	30 (42.3%)
Age (years) Age groups	3 (1–6)
<1 year	2 (2.8%)
1–3 years	36 (50.7%)
4–6 years	19 (26.8%)
≥7 years	14 (19.7%)

### 3.2 Types and locations of foreign bodies

Among the 71 cases, magnetic beads were the most common type (39 cases, 54.9%), followed by coins (9 cases, 12.7%), water beads (6 cases, 8.5%), trichobezoars (4 cases, 5.6%), jujube pits (2 cases, 2.8%), and other rare foreign bodies (10 cases, 14. 1%), including pen caps, lollipop sticks, silicone strips, anti-collision strips, glass fragments, and Ascaris. The distribution of different foreign body types and their locations is shown in Table 2.

**Table 2:** Distribution of foreign body types and retention sites (*n* = 71 patients, 78 retention sites).

Location	Magnetic	Needle	Coin	Water bead	Trichobezoar	Jujube pit	Others	Total
Stomach	6	1	4	0	2	0	7	20
Duodenum	3	0	0	0	0	0	0	3
Colon	3	0	2	0	0	0	0	5
Rectum	1	0	1	0	0	0	2	4
Small intestine	34	0	2	6	2	2	0	46
Total	47	1	9	6	4	2	9	78

Some foreign bodies migrated across multiple locations; the final confirmed retention site was used.

### 3.3 Imaging characteristics

Different types of foreign bodies showed distinct imaging features (Table 3). Magnetic beads and coins were both highly radiopaque on x-ray, with a detection rate of 100% (39/39 for magnetic beads, 9/9 for coins). In contrast, all six cases of water beads were not visualized on plain radiographs and only showed signs of mechanical intestinal obstruction, such as bowel dilatation and air-fluid levels. These cases were ultimately identified by ultrasound and confirmed during surgery.

**Table 3:** Imaging characteristics of different foreign bodies (*n* = 71).

Type	x-ray positivity	Ultrasound features	CT value	US sensitivity (%)	US specificity (%)
Magnetic beads	100%	Beads-like hyperechoic signals + comet-tail artifact	Detect perforation, pneumoperitoneum, ischemia	90.65	100
Coins	100%	Round hyperechoic signal, no shadow	Usually unnecessary	100	100
Water beads	0%	Anechoic cystic structure, thin wall	Detect obstruction, bowel edema, dilation	100	100
Sharp objects	50–80%	Linear hyperechoic signals	Localize perforation or free air	87.5	95.2
Trichobe zoars	Variable	Heterogeneous hyperechoic mass with shadow	Detect large mass and obstruction	75	100

For magnetic foreign bodies, ultrasound demonstrated a sensitivity of 90.65% and specificity of 100% relative to intraoperative findings as the reference standard. Sensitivity varied by location: 96.30% for gastric magnets and 100.00% for small intestinal magnets. For water-absorbing beads, ultrasound achieved a detection rate of 100% (6/6), while plain radiography detected none (0%). For the detection of secondary injury caused by foreign bodies, ultrasound showed a sensitivity of 100% and specificity of 93.5% based on intraoperative confirmation. Interobserver agreement for ultrasound interpretation between the two senior radiologists was substantial, with Cohen's *κ* = 0.78 (95% CI: 0.65–0.91), indicating good reliability of ultrasound findings in this study.

Ultrasound provided complementary diagnostic value: magnetic beads typically appeared as “beads-on-a-string hyperechoic signals with comet-tail artifacts,” while water beads appeared as well-defined, thin-walled anechoic cystic structures. Trichobezoars presented as heterogeneous hyperechoic masses with posterior acoustic shadowing. CT was mainly used to evaluate complications; in 12 cases with suspected perforation or peritonitis, CT successfully detected pneumoperitoneum, bowel wall defects, or intra-abdominal fluid, aiding surgical decision-making.

**Figure 1:**
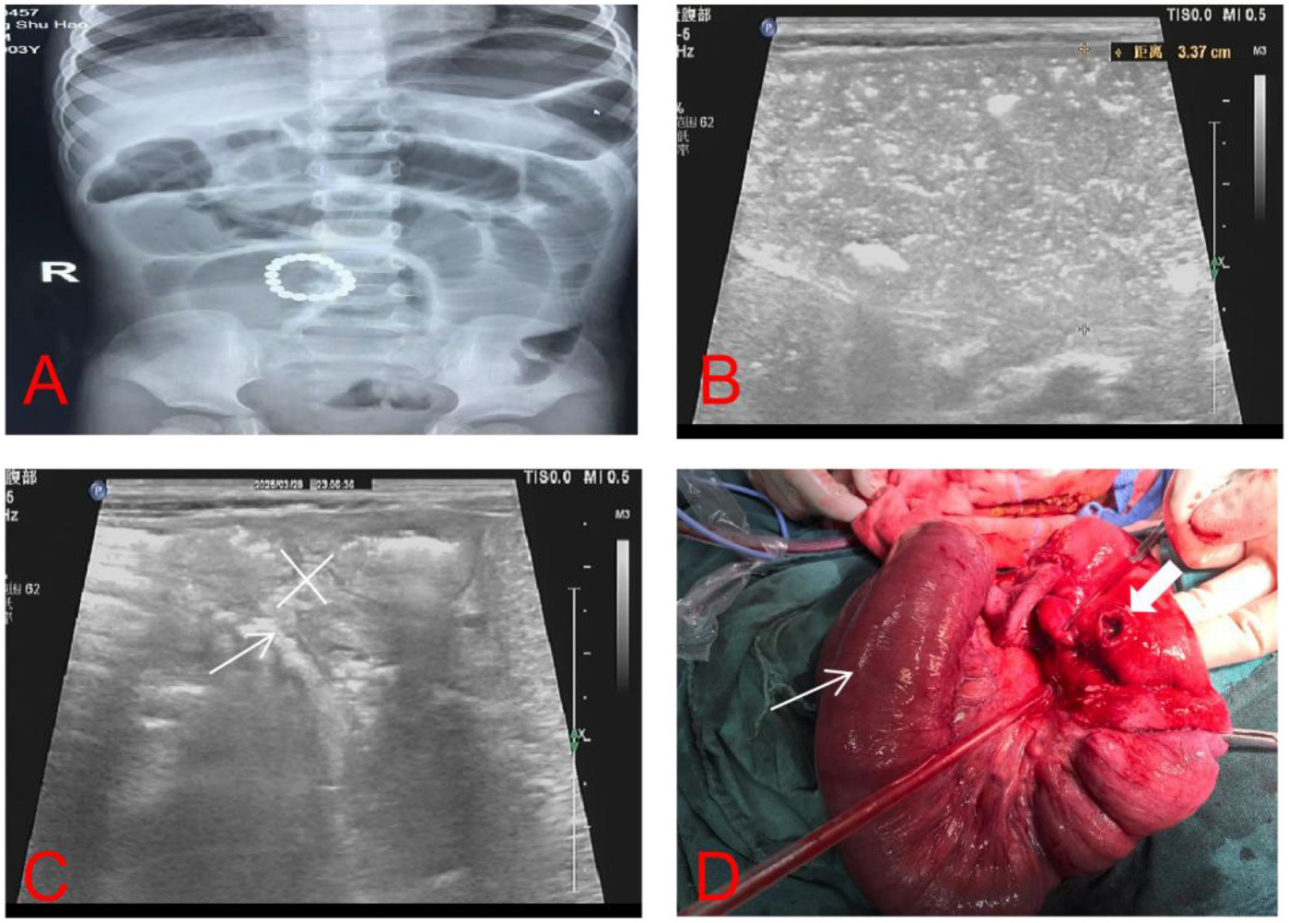
Imaging findings and intraoperative views of intestinal obstruction, internal hernia, and bowel perforation caused by magnetic beads (male child, 3 years old). **(A)** Abdominal x-ray shows partial dilation of intestinal loops in the upper abdomen with air–fluid levels; ring-shaped high-density shadows are seen in the mid–lower abdomen. **(B)** Abdominal ultrasound shows partial dilation of intestinal loops. **(C)** Abdominal ultrasound shows multiple hyperechoic foci with acoustic shadowing (arrows) in the abdominal cavity, along with a “cross-link adhesion” sign. **(D)** Intraoperative findings reveal an internal hernia; part of the intestine is dilated (thin arrow), and bowel perforation is visible (thick arrow).

**Figure 2:**
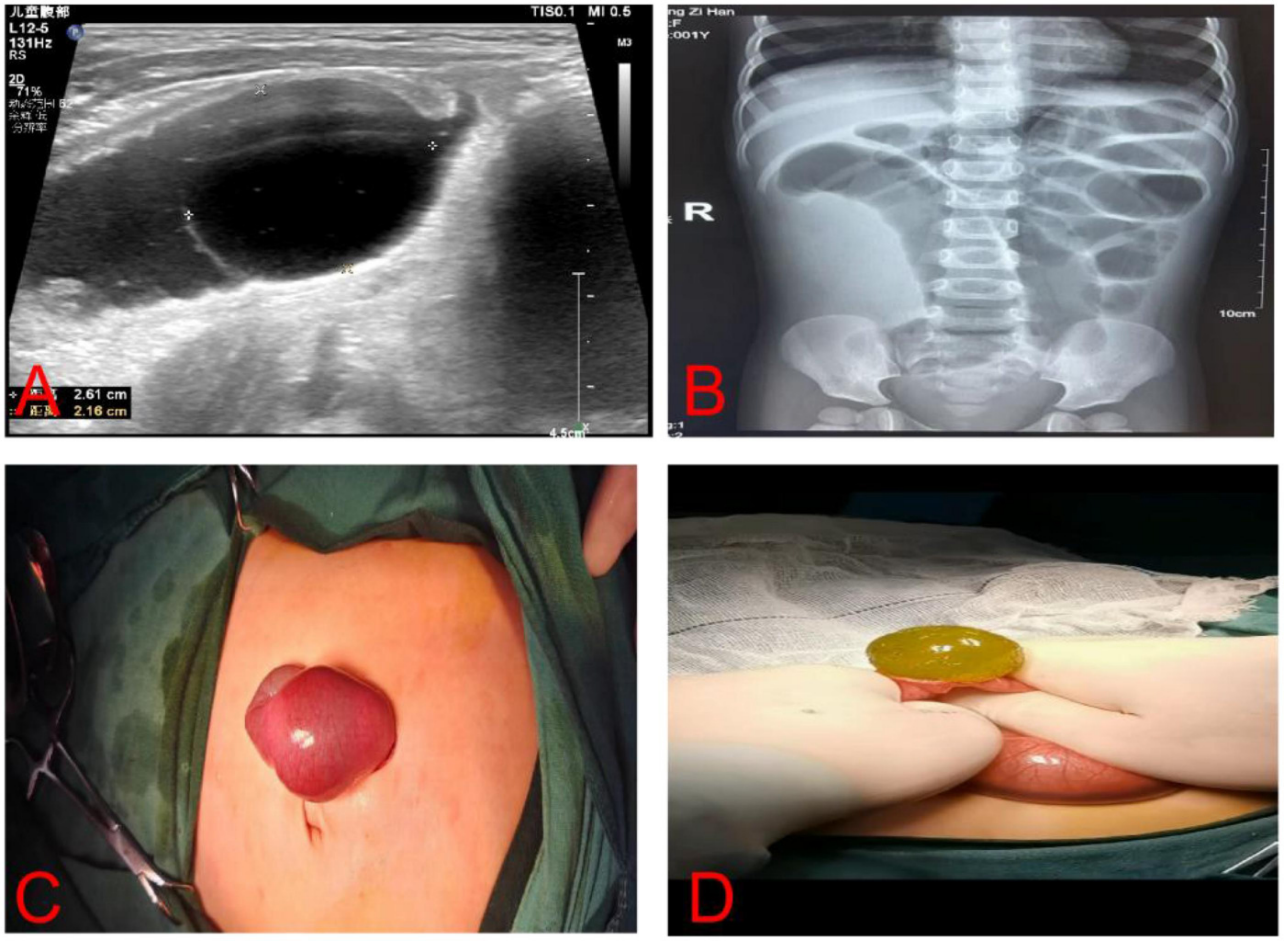
Imaging findings and intraoperative observations of intestinal obstruction caused by a hydrogel bead (female child, 1 year old). **(A)** Ultrasound shows an anechoic area within the intestinal lumen in the right lower abdomen with good acoustic transmission. **(B)** Abdominal x-ray shows no visible foreign body; intestinal gas is present with uneven distribution, along with localized fluid accumulation and air–fluid levels. **(C)** Intraoperative findings show localized dilation of the ileum with a foreign body inside. **(D)** Surgical incision of the intestinal wall reveals a yellow hydrogel bead.

Representative imaging findings are shown in Figures 1,2. Figure 1 shows a 3-year-old boy presenting with intermittent abdominal pain for 7 days, diagnosed with intestinal obstruction, internal hernia, and perforation caused by multiple magnetic beads. Abdominal x-ray showed dilated bowel loops with air-fluid levels and ring-like high-density shadows (Figure 1A). Ultrasound showed bowel dilatation (Figure 1B) and multiple hyperechoic signals with acoustic shadowing forming a “cross-linked adhesion sign” (Figure 1C). Surgery confirmed internal hernia and intestinal perforation (Figure 1D).

Figure 2 shows a 1-year-old girl presenting with vomiting, diagnosed with intestinal obstruction caused by a water bead. Ultrasound revealed a round anechoic structure in the ileum (Figure 2A), while x-ray showed no foreign body but uneven gas distribution and localized air-fluid levels (Figure 2B). Surgery confirmed a dilated ileal segment containing a yellow water bead (Figures 2C,D).

### 3.4 Complications

Among the 71 patients, 50 (70.4%) developed at least one complication, and some had multiple overlapping complications (e.g., perforation with peritonitis or obstruction with adhesions) (Table 4).

**Table 4:** Complications and treatment methods (*n* = 71).

Complication	*n* (%) of patients with complications (*n* = 50)	[Percentage of total cohort (*n* = 71)]	Main associated foreign bodies
Gastrointestinal perforation	32 (64.0)	[45.1]	Magnetic beads (28), magnets (1), magnetic sheet (1), jujube pits (2)
Intestinal obstruction	26 (52.0)	[36.6]	Magnetic beads (16), water beads (6), trichobezoars (2), coins (1)
Intestinal adhesion	17 (34.0)	[24.0]	Secondary to perforation/surgery
Peritonitis	9 (18.0)	[12.7]	Secondary to perforation
Dehydration	6 (12.0)	[8.5]	Due to vomiting
Internal hernia	5 (10.0)	[7.0]	Magnetic objects
≥1 complication	50 (100)	[70.4]	—

Some patients had multiple complications; thus, the sum of percentages exceeds 100%.

(64.0% of patients with complications, 45.1% of the total cohort), including 28 caused by magnetic beads (75.68% of magnetic cases), 1 by a magnet, 1 by a magnetic sheet, and 2 by jujube pits (100% of jujube pit cases). Intestinal obstruction occurred in 26 cases (52.0% of patients with complications, 36.6% of the total cohort), mainly due to high-risk foreign bodies, including water beads (6 cases, 100%), magnetic beads (16 cases, 43.2%), trichobezoars (2 cases), and coins (1 case).

Other complications included intestinal adhesions (17 cases, 34.0% of patients with complications), mostly secondary to perforation or surgery; peritonitis (9 cases, 18.0% of patients with complications), all secondary to perforation; dehydration (6 cases, 12.0% of patients with complications), mainly due to vomiting from obstruction; and internal hernia (5 cases, 10.0% of patients with complications), all caused by magnetic objects.

Severe systemic complications included one case of transient cardiac arrest due to severe dehydration and electrolyte imbalance caused by complete obstruction from a water bead, with full recovery after resuscitation, and one case of septic shock caused by magnetic beads. No deaths occurred in this cohort.

In terms of treatment, 49 cases (69.0%) required surgical intervention, 9 cases (12.7%) underwent endoscopic removal, and 13 cases (18.3%) passed the foreign body spontaneously.

### 3.5 Risk factors for complications

Univariate analysis showed that foreign body type (*P* < 0.001), retention time >24 hours (*P* = 0.003), location in the upper gastrointestinal tract (defined as esophagus or stomach, *P* = 0.012), and presence of symptoms at presentation (e.g., vomiting, abdominal pain, feeding refusal; *P* < 0.001) were significantly associated with complications.

Multivariate logistic regression analysis (Table 5) demonstrated that sharp or corrosive foreign bodies (OR = 5.12, 95% CI: 2.31–11.34), retention time >24 hours (OR = 4.87, 95% CI: 2.05–11.58), location in the upper gastrointestinal tract (OR = 3.21, 95% CI: 1.42–7.26), and symptomatic presentation (OR = 3.94, 95% CI: 1.78–8.72) were independent risk factors (all *P* < 0.01). The Hosmer–Lemeshow goodness-of-fit test yielded *P* = 0.32, indicating good model fit.

**Table 5:** Multivariate logistic regression analysis of risk factors for complications (*n* = 71).

Variable	*β*	OR (95% CI)	*P* value
Sharp or corrosive foreign bodies	1.633	5.12 (2.31–11.34)	<0.001
Retention time >24 h	1.583	4.87 (2.05–11.58)	<0.001
Upper gastrointestinal location	1.167	3.21 (1.42–7.26)	0.005
Symptomatic at presentation	1.372	3.94 (1.78–8.72)	0.001
[Model 2: Magnetic beads as independent predictor]	1.861	6.43 (2.87–14.41)	<0.001

Hosmer–Lemeshow goodness-of-fit test *P* = 0.32. Model 1 (presented above) included the composite variable “sharp or corrosive foreign bodies.” Model 2 (shown in brackets) replaced this with “magnetic beads” as an independent categorical predictor.

In a separate multivariate model in which magnetic beads were entered as an independent categorical predictor instead of being merged into the sharp/corrosive category, magnetic beads also independently predicted complications (OR = 6.43, 95% CI: 2.87–14.41, *P* < 0.001), after adjusting for retention time, location, and symptomatic presentation. This finding confirms that multiple magnetic beads carry a risk of complications that is comparable to—and in this cohort even exceeded—that of traditional sharp objects.

Notably, although magnetic beads are traditionally classified as “blunt objects,” their tendency for multiple ingestion and magnetic attraction can lead to compression between adjacent bowel loops, resulting in ischemic necrosis. Their perforation rate (59.5%) was significantly higher than that of coins (0/12) and comparable to that of sharp objects. Therefore, in this study, multiple magnetic beads were categorized as high-risk foreign bodies in clinical risk stratification.

## 4 Discussion

This study retrospectively analyzed 71 cases of pediatric gastrointestinal foreign bodies. The results showed that most patients were males aged 1–3 years (50.7%), which is consistent with previous reports identifying this age group as having the highest incidence of foreign body ingestion (1, 9). Notably, magnetic beads accounted for 54.9% (39/71) of cases, making them the predominant type. This distribution differs from earlier reports in which coins were most common (1), but aligns with recent international studies, suggesting that magnetic toys have become a new focus in pediatric gastrointestinal foreign body prevention and management (5, 10).

Comparison with previously reported epidemiological data provides important context. A large single-center study of 614 pediatric cases reported complications in 152 children (24.8%), with gastrointestinal perforation in 6.2% (12). In contrast, the overall complication rate in the present cohort was 70.4%, and the perforation rate was 45.1%. This discrepancy is primarily attributable to the high proportion of magnetic bead ingestion (54.9% vs. 8.5% in the comparative study), highlighting the elevated risk associated with magnetic toys. Another prospective study reported that over 80% of ingested foreign bodies pass spontaneously without intervention (10); however, only 13 patients (18.3%) passed spontaneously in our series, while 49 (69.0%) required surgery. These findings underscore that the type of foreign body strongly determines clinical outcomes, and that the rise of magnetic toys has transformed the risk profile of pediatric foreign body ingestion.

This study highlights the unique hazards of magnetic beads and water-absorbing beads. Traditional risk stratification considers smooth, blunt objects (e.g., coins) as low-risk (2). However, although magnetic beads are morphologically blunt, their perforation rate in this study reached 71.8% (28/39), challenging conventional classification based solely on shape. The core mechanism is pressure necrosis caused by magnetic attraction: when multiple magnets reside in different bowel loops, strong trans-wall forces cause sustained compression, ischemia, necrosis, and eventual perforation (5, 10). Ultrasound findings such as the “string-like hyperechoic pattern with comet-tail artifact” and the “cross-linked adhesion sign” reflect this magnetic interaction. These findings are consistent with studies by Litovitz et al. and Naomi et al., who identified multiple magnetic foreign bodies as a major risk factor for intestinal perforation, with significantly higher risk than single magnets or other blunt objects (5, 12). Notably, one patient who ingested 48 magnetic beads developed multiple perforations in the stomach and colon, confirming that a higher number of foreign bodies exacerbates injury severity.

All six cases of water bead ingestion resulted in intestinal obstruction (100%). The mechanism involves rapid expansion of water-absorbing polymers, which increase exponentially in volume and cause mechanical obstruction, particularly in the narrow terminal ileum (6, 13). Ultrasound showed well-defined, thin-walled anechoic structures, whereas x-ray failed to detect these objects, explaining why such patients often present with “unexplained obstruction” until diagnosis is confirmed by ultrasound or surgery. Gatto et al. similarly emphasized that delayed diagnosis of radiolucent foreign bodies significantly increases the risk of obstruction and intestinal ischemia (14). Regarding button batteries, although only one case was included in the present cohort, a systematic review of 439 pediatric button battery exposures reported common complications including esophageal mucosal damage (26.3%) and tracheoesophageal fistula (23.3%), with intestinal perforation in 2.5% (11). Age ≤2 years, exposure >6 hours, and battery diameter ≥20 mm were identified as independent predictors of severe complications. The single battery case in our series resulted in lower gastrointestinal perforation, consistent with this time-dependent injury. However, given the small number of battery cases, the OR estimates for the composite “sharp or corrosive” category in this study are primarily driven by magnetic beads and jujube pits, and caution is warranted in generalizing these estimates to button batteries specifically.

Retention time >24 hours was identified as an independent risk factor. The pathophysiology involves cumulative injury and progressive inflammatory response. Whether from sharp objects, electrochemical corrosion from batteries, or sustained compression from magnets, mucosal injury worsens over time (15). In this study, retention >24 hours increased complication risk nearly fivefold (OR = 4.87). For example, button batteries can cause mucosal burns within 2 hours and deep perforation within 4–6 hours when lodged in the esophagus (7). For magnetic foreign bodies, prolonged retention allows more time for pressure-induced ischemia and necrosis. A multicenter study also identified the time from ingestion to intervention as one of the strongest predictors of complications in magnetic foreign body cases (16). Our findings reinforce current guidelines recommending emergency management of high-risk foreign bodies and highlight the critical importance of timely intervention (17).

Upper gastrointestinal tract location (esophagus/stomach) was another independent risk factor, with a 3.21-fold higher complication risk compared to lower tract. The esophagus, particularly at its three physiological narrowings, has delicate mucosa and limited mobility. Once a foreign body becomes lodged, it can cause significant direct injury and is less likely to pass spontaneously due to local edema, leading to prolonged retention and a vicious cycle of damage (18). In this study, both cases of esophageal jujube pits resulted in perforation. For gastric foreign bodies, the acidic environment may accelerate electrolyte leakage from batteries, and gastric peristalsis may promote aggregation of magnetic objects, increasing the risk of hazardous configurations. These findings are consistent with Li et al., who reported a significantly higher complication rate for upper gastrointestinal foreign bodies (3). However, most magnetic beads (34/39) in this study were retained in the small intestine, where perforations also frequently occurred. This suggests that risk is not determined solely by location but also by interaction with foreign body characteristics. The small intestine, with its thin wall and high mobility, is particularly susceptible to compression between magnetic beads and should also be considered a “high-risk site” for magnetic foreign bodies.

In this study, symptomatic patients had a 3.94-fold higher risk of complications than asymptomatic patients. Symptoms such as vomiting, abdominal pain, and feeding refusal reflect underlying mucosal irritation, luminal obstruction, or wall injury caused by the foreign body (19). All patients with intestinal obstruction presented with abdominal pain and vomiting, while those with esophageal foreign bodies commonly exhibited drooling and dysphagia. These symptoms indicate a transition from a “silent” to an “injurious” state, requiring heightened clinical vigilance. This finding is consistent with previous studies that identified clinical symptoms as independent predictors of poor outcomes (20). Importantly, while symptoms are not a causal risk factor in the strict sense, they are valuable clinical warning signs that should prompt immediate diagnostic evaluation and consideration of intervention.

The high incidence of water bead and jujube pit ingestions in the present cohort warrants comment. Water beads have gained popularity as children's sensory toys in East Asian markets, and their ingestion has been increasingly reported in Chinese pediatric hospitals. Similarly, jujube pits—the hard seeds of jujube dates, which are commonly consumed as a snack in China—are rarely encountered in European or North American practice. Their absence in European cohorts reflects cultural and dietary differences in the objects accessible to children. Conversely, magnetic beads appear to be a global problem, with rising incidence reported worldwide, likely due to the widespread availability of high-powered magnetic toy sets (21). These regional variations underscore the importance of context-specific prevention strategies and clinical awareness.

Based on the imaging performance data, we propose that ultrasound should be employed as a complementary first-line imaging modality in specific scenarios. For suspected radiolucent foreign bodies (e.g., water beads, plastics, wood), ultrasound should be performed even if plain radiography is negative. For magnetic foreign bodies, ultrasound offers excellent sensitivity (90.65%) and specificity (100%) for detection and localization (22), with the added advantage of real-time assessment of bowel wall integrity and detection of the “cross-link adhesion sign” indicative of trans-mural attraction. Given the substantial interobserver agreement in this study (*κ* = 0.78), ultrasound findings are reproducible between experienced operators.

From a management perspective, the independent risk factors identified—sharp/corrosive foreign bodies (including multiple magnetic beads), retention time >24 hours, upper gastrointestinal location, and symptomatic presentation—should guide clinical decision-making. Patients with any of these high-risk features warrant early endoscopic or surgical intervention rather than conservative observation. In particular, children with multiple ingested magnetic beads should be managed as high-risk regardless of the absence of initial symptoms, as the insidious development of pressure necrosis may precede clinical deterioration.

## 5 Limitations

This study has several limitations. As a single-center retrospective study, the sample size was relatively small, and the number of certain high-risk foreign bodies (e.g., button batteries) was limited, which may affect precise risk estimation. Specifically, the button battery group contained only one patient, and the sharp object group contained only five patients; therefore, the OR estimates for the composite “sharp or corrosive” category are heavily weighted by magnetic bead cases. Caution is warranted in generalizing these estimates to button batteries specifically. The retrospective design also limits standardization of imaging timing and protocols. Furthermore, this study did not perform long-term follow-up to assess late complications such as adhesive bowel obstruction following surgery, which may occur months or years after the initial event. Future multicenter, prospective studies with larger sample sizes and extended follow-up are needed for validation.

## 6 Conclusions and clinical implications

The spectrum of pediatric gastrointestinal foreign bodies has shifted significantly. Multiple magnetic beads and water-absorbing beads now represent leading causes of severe complications. In this study, magnetic beads were associated with a perforation rate of 71.8%, and water beads caused intestinal obstruction in 100% of cases—far exceeding the risks posed by traditional blunt objects such as coins. These findings support classifying both multiple magnetic beads and water beads as “extremely high-risk” foreign bodies in clinical practice.

Imaging strategies should be optimized accordingly. While plain radiography remains effective for metallic objects, it fails to detect radiolucent materials like water beads. Ultrasound plays an irreplaceable role in identifying magnetic beads (via comet-tail artifacts), water beads (as anechoic structures), and early complications such as bowel ischemia or adhesions. It should be routinely employed as a complementary first-line modality in x-ray-negative but clinically suspicious or high-risk cases.

Four key factors independently predicted complications: (1) ingestion of multiple magnetic beads, (2) sharp or corrosive foreign bodies (e.g., jujube pits, button batteries), (3) retention time exceeding 24 hours, (4) location in the upper gastrointestinal tract, and (5) presence of clinical symptoms at presentation. Patients exhibiting any of these features warrant prompt endoscopic or surgical intervention rather than conservative observation. The strong independent association between multiple magnetic bead ingestion and complications (OR = 6.43) underscores the need to reclassify these objects as “extremely high-risk,” despite their morphologically blunt appearance.

## Data Availability

The original contributions presented in the study are included in the article/Supplementary Material, further inquiries can be directed to the corresponding author.
